# Population-genetic properties of differentiated copy number variations in cattle

**DOI:** 10.1038/srep23161

**Published:** 2016-03-23

**Authors:** Lingyang Xu, Yali Hou, Derek M. Bickhart, Yang Zhou, El Hamidi abdel Hay, Jiuzhou Song, Tad S. Sonstegard, Curtis P. Van Tassell, George E. Liu

**Affiliations:** 1 Animal Genomics and Improvement Laboratory, Agricultural Research Service, USDA, Beltsville, Maryland 20705, USA; 2 Department of Animal and Avian Sciences, University of Maryland, College Park, Maryland 20742, USA; 3 Beijing Institute of Genomics, Chinese Academy of Sciences, Beijing, 100101, China; 4 College of Animal Science and Technology, Northwest A&F University, Shaanxi Key Laboratory of Agricultural Molecular Biology, Yangling, Shaanxi, 712100, China

## Abstract

While single nucleotide polymorphism (SNP) is typically the variant of choice for population genetics, copy number variation (CNV) which comprises insertion, deletion and duplication of genomic sequence, is an informative type of genetic variation. CNVs have been shown to be both common in mammals and important for understanding the relationship between genotype and phenotype. However, CNV differentiation, selection and its population genetic properties are not well understood across diverse populations. We performed a population genetics survey based on CNVs derived from the BovineHD SNP array data of eight distinct cattle breeds. We generated high resolution results that show geographical patterns of variations and genome-wide admixture proportions within and among breeds. Similar to the previous SNP-based studies, our CNV-based results displayed a strong correlation of population structure and geographical location. By conducting three pairwise comparisons among European taurine, African taurine, and indicine groups, we further identified 78 unique CNV regions that were highly differentiated, some of which might be due to selection. These CNV regions overlapped with genes involved in traits related to parasite resistance, immunity response, body size, fertility, and milk production. Our results characterize CNV diversity among cattle populations and provide a list of lineage-differentiated CNVs.

Copy number variations (CNVs) are large-scale insertions and deletions, existing as one type of complex multiallelic variants within diverse populations^1^,^2^. Compared to single nucleotide polymorphisms (SNPs), CNVs involve more genomic sequences and have potentially greater effects, including changing gene structure and dosage, altering gene regulation and exposing recessive alleles^3^. Human and mouse studies found that CNVs captured 18–30% of the genetic variation in gene expression^4^,^5^. These CNVs were shown to be important in both normal phenotypic variability and disease susceptibility. Population genetics has played an important role in exploring genetic variations in human^6^ and farm animals^7^. Investigating the population genetics and evolutionary origins of CNVs could enable us to understand their origins and impacts^8^,^9^,^10^,^11^. With recent advances in our knowledge of the locations, sizes and mutational mechanisms of CNV using high-throughput screening approaches, the attempt to study corresponding population genetics is gradually developing in human and other model species. Findings from these initial studies have brought new insights into genome diversity and adaptation^12^,^13^,^14^,^15^.

Population structure analyses based on human CNVs have revealed results largely consistent with those based on SNPs of similar number^16^. For instance, based on hybrid genotyping arrays, up to 90% of human CNVs can be revealed by integrated investigation of SNPs^17^. On the other hand, multiple lines of evidence also suggest CNVs could serve as an extra genomic resource and provide important insights into the origins and sub-structure of populations^9^,^15^,^16^,^18^,^19^,^20^,^21^,^22^. Additionally, population-specific CNVs are candidate regions under selection and are potentially responsible for diverse phenotypes^9^,^23^,^24^.

Previous studies have also revealed that genomic diversity could be generated by the bias of selection on CNV in specific environments for adaptations^25^. For instance in human adaptations, positive selection for a higher *AMY1* copy number enables the better digestion of starchy foods^26^. An indel polymorphism in gene *APOBEC3b* has been associated with malaria susceptibility^27^. The human *UGT2B17* gene shows significant copy-number diversity among populations from Africa, Europe, and East Asia, which displays region-specific differences in the metabolism of steroid hormones and a large number of xenobiotics^28^. Another well-known example is the olfactory receptor (OR) genes, which are frequently found to be copy-number variable in most mammalian species. The differences in OR gene counts between human populations suggested that they are involved in population-specific differences in smell^29^. In addition, CNVs are specifically enriched among evolutionary “young” ORs, implying that CNVs may play a critical role in the processes of gene birth and death or the emergence of new OR gene clusters^30^.

In livestock, such as cattle, most CNV studies have limited themselves to CNV detection and enumeration using various platforms, such as CGH array, SNP array or next generation sequencing^31^,^32^,^33^,^34^,^35^,^36^,^37^,^38^,^39^. Even though the aforementioned studies have identified a large number of copy number variable regions in their respective species, exploring livestock population genetics using cattle CNVs is still in its infancy. The investigation of diversity and origin of CNVs, the characterization of their population-genetic properties, and the determination of the functional impacts of CNVs are still active areas of research.

Here, we report a comprehensive population-genetics study of CNVs by focusing on the diversity, population structure, and selection of identified CNVs within eight representative cattle breeds. In this study, we investigated CNVs from individuals originating from European taurine, indicine, and African taurine breeds of the Bovine HapMap DNA panel^40^. Our results revealed that most common CNVs, especially CNV deletions, show large differences in frequency across diverse groups. More importantly, we demonstrated that CNVs can be used for the investigation of population genetics in cattle, as we observed CNVs with significant diversity across groups that might be associated with breed and sub-species specific selection signatures.

## Results

### CNVs segmentation and genotyping

A total of 300 individuals was used for CNV discovery as shown in Table S1, including Holstein (HOL), Angus (ANG), Hereford (HFD), Brown Swiss (BWS), Brahman (BRM), Nelore (NEL), N’Dama (NDA), and Sheko (SHK). In total, 155,700 CNV segments were extracted by Golden Helix SVS 8.0 using the default multivariate option. After merging across all individuals, we discovered 263 non-redundant CNVs which are commonly shared within the whole population (Table S2). Since the SVS multivariate option was developed to identify moderate to high frequency CNVs, only segments with frequencies above 1% were retained for further analysis in order to filter away potential false positive calls. Finally, a total of 257 CNVs (with a total length of 12,444 kb and an average length of 48.4 kb) were retained and used to categorize the samples as one of three types (loss, neutral and gain events) according to a three-state model with strict threshold levels of marker mean log R ratio (LRR) ± 0.3. They were sorted as a list of CNV1 to CNV257 with a descending frequency, in which there were 184 deletion CNVs (Table S2). As shown previously^41^,^42^, comparisons of CNV detection algorithms usually revealed a low concordance. However, when we compared this dataset with our previous results using PennCNV in the same Bovine Hap Map samples^32^, we obtained a total of 160 concordant CNVs (61%), indicating a high quality of our SVS results. While all 257 CNVs were used for frequency and *V*_*ST*_ calculations, only the 184 deletion CNV regions were used in all other subsequent population genetics analyses.

### Population-genetic properties of cattle CNVs

#### Hierarchical Clustering Analysis

**Figure 1:**
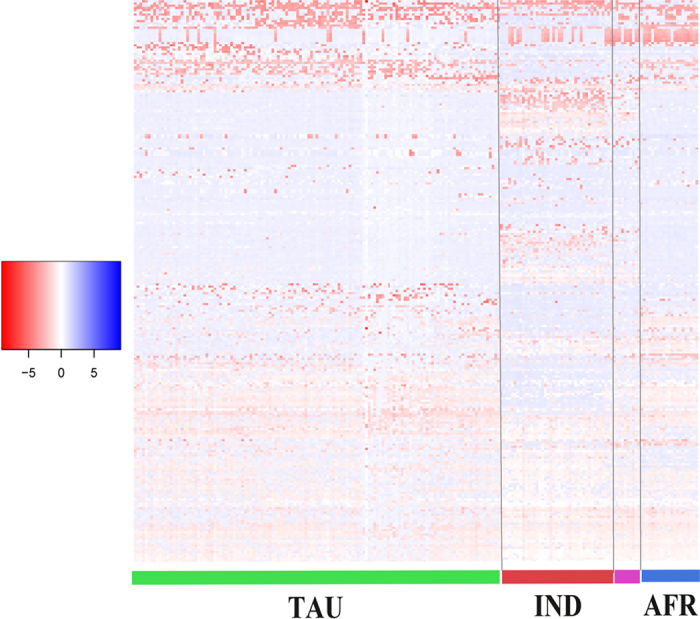
Eight diverse cattle breeds were grouped into four clusters in this heatmap based on the mean segment LRR of 257 CNVs, including European taurine in green (TAU), indicine in red (IND), and African taurine in blue (AFR). As expected, SHK in pink was located between AFR and IND. Cattle were clustered horizontally according to their breeds, and CNV were vertically arranged by the clustering method.

To obtain a global picture of group differences, hierarchical clustering was done using the mean LRRs for the 257 CNVs. Three distinct groups were observed, including group one European taurine (TAU) containing HOL, ANG, HFD, and BWS; a second indicine (IND) group containing BRM and NEL; and third group African taurine (AFR) containing NDA (Fig. 1). SHK, which used to be considered a taurine population, because they are humpless, was positioned between IND and AFR confirming SHK is a hybrid breed in agreement with its known breed formation history^43^.

#### Multidimentional Scaling (MDS) Analysis

**Figure 2:**
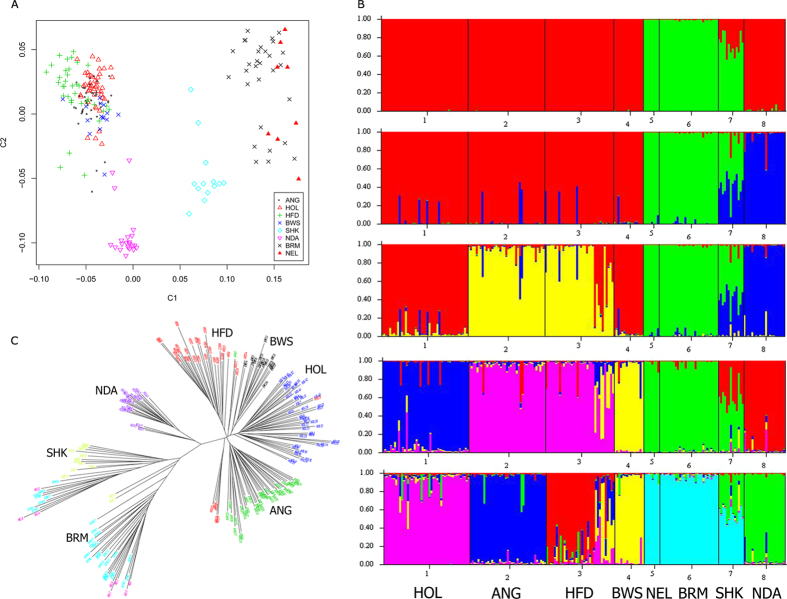
Population genetic analyses of eight diverse cattle breeds based on 184 deletion CNVs. Four distinct groups include the European taurine (TAU) group containing HOL, ANG, HFD, and BWS; the second indicine (IND) group containing BRM and NEL; the third group African taurine (AFR) containing NDA; and the fourth group formed by the hybrid SHK. (**A**) MDS analysis of 205 individuals. Individuals were plotted according to their coordinates on the first two components. (**B**) Clustering of 205 individuals from eight breeds based on 184 deletion CNVs when K = 2–6. Individuals were shown as a thin vertical line colored in proportion to their estimated ancestry. (**C**) Neighbor-joining tree of the 205 individuals. The tree was constructed using genetic sharing distances. Edges were labeled according to the breed of origin.

To examine the population structure of these three cattle groups based on CNVs, a multidimensional scaling (MDS) analysis was completed on 205 unrelated individuals based on the 184 deletion CNVs (Fig. 2A). We found C1 axis can clearly separate taurine (TAU and AFR) from indicine (IND), while C2 axis can separate African taurine (AFR) into its unique cluster with a small amount of intermixing with European taurine (TAU). Therefore, the global organization of cattle genetic diversity can be represented as a triangle with apexes corresponding respectively to TAU, IND, and AFR groups. As expected, we observed that SHK was located between AFR and IND, again confirming its hybrid breed formation history. We found this CNV-based MDS results are generally consistent with the results from a similar SNP-based analysis^40^,^44^, suggesting CNVs can be used to separate cattle individuals into distinct groups. However, the clustering resolution within groups based on CNVs was not better than those based on SNPs. For example, CNVs cannot distinguish the HOL breed from the ANG breed in European taurine cattle. There were also certain degrees of mixing within indicine individuals in the CNV-based clustering results (Fig. 2A). In summary, our results revealed that CNV can be used in population genetic studies. However, compared to SNP, CNV suffers from small sampling size and difficulty to genotype, making it difficult to use them to do fine clustering, especially within a group.

#### Admixture Analysis

To investigate genome wide ancestral admixture patterns of these eight breeds, we used the admixture inference method implemented in STRUCTURE (Fig. 2B). Varying the number of presumed ancestral populations (K) recapitulated the extent of genetic divergences across breeds. At K = 2, TAU and AFR were clearly assigned into unique groups distinct from IND. At K = 3, the clustering analysis revealed TAU was separated from AFR showing a clear separation of TAU, IND and AFR groups. At K = 4, intriguingly, European taurine beef breeds was separated from their dairy counterparts. At K = 5, BWS was deviated from HOL. Finally at K = 6, HFD was separated from ANG and most of the samples were clustered according to breed designation, except that the NEL and BRM breeds were still clustered together. In addition, increasing the number of inferred clusters allowed us to confirm a high level of admixture and support the documented origin of SHK, which accommodated high fractions of admixture from ancestries of AFR and IND. Overall, these results were in agreement with our MDS analysis, suggesting that the partitioning of cattle into distinct populations is closely related to genetic diversity, which is in agreement with the earlier report by Bovine HapMap Consortium^40^.

#### Neighbor-Joining Clustering Analysis

In addition, we calculated all pairwise genetic distances using PLINK 1.07, and plotted a neighbor-joining dendrogram of all individuals (Fig. 2C). We found that the genetic relationship among cattle groups could be largely recovered from this dendrogram as it clearly arranged individuals according to their population of origin. Although two indicine breeds (BRM and NEL) are intermixed, the three breed groups (TAU, AFR and IND) can be easily distinguished. In agreement with MDS and admixture results, individuals from SHK branched between AFR and IND. This clustering analysis of individual samples supports most of the relationships among the cattle breeds uncovered by our MDS and STRUCTURE analysis.

### Population diversity and differentiated CNVs

Using 257 CNVs, we estimated the CNV frequencies across 3 cattle groups, i.e. TAU, IND and AFR. With an average of frequency of 0.39, we indentified the top five high frequency deletions were on chromosomes 11, 26, 4, 29, and 11 with the corresponding frequencies of 0.93, 0.93, 0.92, 0.92, and 0.90, respectively (Table S2). Using the 205 unrelated individuals, we estimated the frequencies for each group to investigate CNVs with differentiated frequencies.

We compared CNV regions with the UMD 3.1 Ensembl gene (EnsGene) annotation and found 101 CNVs partially overlapping with genes (Table S3). We performed gene ontology (GO) analysis using PANTHER^45^ to identify if there was enrichment of genes with specific function (Table S4). The most enriched biological processes include response to interferon-gamma (Interferon-Induced Guanylate-Binding Protein 2), other immunity related processes, and response to stimulus (MHC, immunoglobins, ORs and ATP-binding cassette (ABC) transporters). Aside from 99 CNVs that overlapped with EnsGene genes, we found 158 CNVs which did not encompass any EnsGene genes. When comparing CNVs overlapped with genes to CNVs not overlapped with genes, we found that the CNVs without genes were shorter (with Mean 23370 ± Standard Error of Mean 5659, N = 158 vs. 88400 ± 18500, N = 99, t-test, p-value < 0.0001) and at higher frequency (0.4218 ± 0.0189, N = 158 vs. 0.3468 ± 0.0218, N = 99, p-value = 0.0113). The paucity of common deletion CNVs overlapping with genes is consistent with the notion that they are under purifying selection, which removes deleterious variants from the population. We noted that our length and frequency analysis could be confounded if the power to detect short events is higher than long events and the power to detect common deletions is higher than common duplications. Besides other neutral possibilities, like those indicated in an early human study^14^, CNVs may also affect gene expression through regulatory level changes.

**Figure 3:**
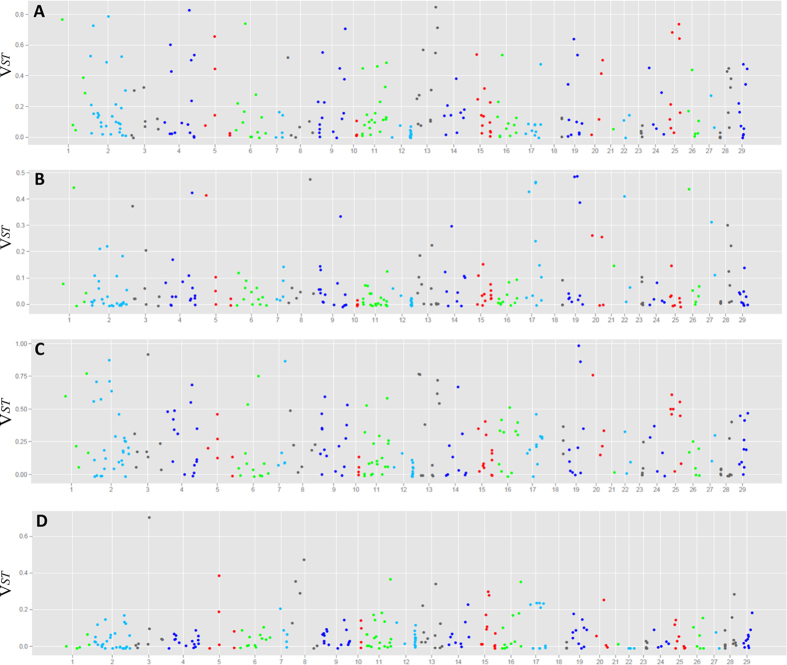
Genome wide *V*_*ST*_ value plots for CNVs in the following comparisons: (**A**) TAU vs. IND; (**B**) TAU vs. AFR; (**C**) IND vs. AFR; and (**D**) HOL vs. ANG.

CNVs that differ greatly in frequency between cattle groups/breeds are candidates for population-specific selection. To test whether any CNV might be associated with population-specific selection, we estimated the pairwise *V*_*ST*_ for 4 comparisons, including TAU vs. IND, TAU vs. AFR, IND vs. AFR, and HOL vs. ANG (Fig. 3, Fig. S1, and Table S3). *V*_*ST*_ estimations produce values from 0 (no difference) to 1 (complete population differentiation), with high *V*_*ST*_ values indicating regions under increased selective pressure or other evolutionary forces, such as bottlenecks or founder effects. Using a stringent threshold cutoff of *V*_*ST*_ > 0.6, we detected a total of 14, 0, 18, 1 CNV(s) in the abovementioned four comparisons, respectively. When we lowered the threshold to 0.4, we observed 41, 11, 48, 2 CNVs for the four comparisons, respectively.

The higher differential *V*_*ST*_ identified in these CNV regions may suggest the dosage variability of their underlying genomic sequence, which could be further involved in the diverse phenotypes across cattle breeds. For instance, when comparing TAU with IND under the lower threshold of 0.4, we observed ten genes overlapped with CNV regions, including *CDH18*, *GDAP1L1*, *HIATL1*, *IGLL1*, *ITGB8*, *KCNIP3*, *LCT*, *NETO1*, *OIT3*, and *SHISA9*. Similarly for the comparisons of TAU vs. AFR and IND vs. AFR, we found nine genes (*EPHB3, FANCC, GRM7*, *HSFY2*, *KCNJ12*, *LIPF*, *PRAME*, *TSPY*, and *ZNF280B*) and nine genes (*GDAP1L1*, *HIATL1*, *LCT*, *MRPL48*, *MSMB*, *PLCB1*, *RBFOX1*, *ROBO4*, and *SHISA9*) overlapping with CNV regions, respectively. Although for some genes, only small parts were covered by CNVs, the change of these small regions could potentially influence their function and evolution. We further overlapped these genes with the known cattle quantitative trait locus (QTL at http://www.animalgenome.org/cgi-bin/QTLdb/BT/index) and found genes associated with important traits in cattle that vary in copy number frequencies across populations (Fig. S2). For example, some of them are related to parasite resistance, immunity response and adaption, including *EPHB3*, *SHISA9* and *LCT*^46^,^47^. Other genes were reported to be involved in body size, fertility, production and milk fatty acid profile, for examples *FANCC*, *IGLL1* and *LIPF*^48^,^49^.

## Discussion

Population genetics studies based on CNVs have been explored in human, dog, zebrafish, and stickleback fish^8^,^9^,^23^,^50^. Despite that previous studies have identified CNVs within and between populations in cattle, our study is one of the first attempts to explore the population-genetic properties in cattle based on CNVs derived from the high-density SNP array. We also provided additional evidence to support CNVs as genetic markers that can be used to study the across population diversity and capture the subspecies relationships. Since it was difficult to accurately detect and genotype complex CNV events, like non-biallelic duplications, in this proof-of-principle study, we mainly used high confidence deletion CNVs. The distinct advantage of deletion CNVs over duplication events is that deletions can be treated as bi-allelic markers, and are therefore compatible with mature genetics analysis methods designed for SNP markers.

In the current study, we used CNVs with moderate intra-population frequencies to explore the population-genetic properties in cattle. Although the SVS method we utilized reported a limited amount of CNV calls, including 71.6% deletions and 28.4% duplications, our study did reveal that globally diverse cattle populations clustered roughly by geographical region and were influenced by demographic history, which is similar to the results derived from previous SNP-based cattle studies^40^,^51^,^52^. This could be due to the fact that 75% of the CNVs that we identified were well tagged by flanking SNPs, as was estimated previously^53^. We also found the results of this survey were not capable of distinguishing recently divergent cattle breeds of common geographic origin, such as HOL and ANG breeds, which were reported to be separated by 364 generations^54^. This observation was consistent with human studies, where published reports also showed the CNV-based population stratification can be only detected among the large population groups at the continent level^15^. The fine genetic structure detected by SNPs may be due to their accuracy of genotyping and sample size, besides other influencing factors, as suggested previously^16^. Further study based on high throughput sequencing will make it easier to accurately genotype CNVs^11^,^34^.

To investigate lineage-differentiated CNVs in the cattle genome, we also conducted CNV-based population differentiation analysis, and identified potential CNV candidates under divergent selection. We estimated *V*_*ST*_ values, a population differentiation estimator similar to *F*_*ST*_, among groups and examined gene enrichments among CNVs regions. In our previous study based on array CGH in cattle populations, we revealed that regions that have been under recent positive selection exhibit elevated population differentiation^33^. In the current study, we found 78 unique CNVs with *V*_*ST*_ values above the threshold of 0.4 as potential lineage- differentiated events in three group comparisons, perhaps representing increased selective pressures exerted upon the cattle population. It is noted that besides selective pressure, the amount of divergence between populations (time since divergence, effective population size, and gene flow/migration) also can affect the overall differentiation and *V*_*ST*_ values. High *V*_*ST*_ between groups does not necessarily involve divergent selection (selection in both populations for different alleles), and can also occur in the absence of selection, for example, by bottlenecks or founder effects followed by drift. All these hypotheses warrant further investigations using larger sample sizes.

By contrast, we observed only a handful of lineage-differentiated CNVs in our HOL vs. ANG comparison, which did not overlap with any known cattle genes. Fewer lineage-differentiated CNVs may suggest that these two breeds might not have had sufficient time to diversify their deletion CNV contents, if we assume that the CNV occurred before the split of the two breeds, and that the deletion event should eventually fix in one but not the other population. Additionally, fewer lineage-differentiated CNVs were also observed in laboratory mouse and zebrafish strains, as compared with their wild populations^23^,^55^. This may be indicative of either inbreeding effects or suggest that CNVs were preferentially fixed as a consequence of the larger effective population size of wild populations. It is well known that HOL and ANG have gone through intensive human selection recently via the practice of artificial insemination.

In the future, we propose that additional cattle breeds from places like the Middle East, Pakistan and Turkey will provide more insights into the worldwide and local genetic diversity and population structure. We also expect that discovery of low frequency CNV variants, especially in those under-represented breeds like indicine and African taurine cattle will provide additional resolution for distinguishing those populations. Finally, with more powerful software tools^56^, we predict population genetics in livestock will remarkably expand with next generation sequencing data.

## Methods

### Samples

In the CNV discovery phase, we retrieved a subset of Illumina BovineHD SNP dataset (300 individuals, Table S1), which represent 8 geographically diverse breeds, including Holstein, Angus, Hereford, Brown Swiss, N’Dama, Sheko, Brahman, and Nelore^32^. All chosen samples had a genotyping success rate of more than 99%. For population genetic analyses, we only used 205 animals after removing related individuals according to pedigree information and pi-hat value if it was more than 0.4.

### CNV segmentation and genotyping

The intensity data of 742,910 SNP probes were generated using the Illumina BovineHD SNP array. After exporting the DSF file from GenomeStudio Software, we imported Log R Ratio (LRR) into Golden Helix SNP & Variation Suite (SVS) 8.0 (Golden Helix Inc., Bozeman, MT, USA) and successfully mapped 735,293 SNPs (98.97%) onto the 29 autosomes of *Bos taurus* genome assembly UMD 3.1. The LRR was then normalized using the default GC correlation file to correct the waviness caused by the GC content. We then utilized the copy number analysis module (CNAM) under the multivariate option to segment chromosomes with default set, and a significance level of p = 0.01 for pairwise permutations (n = 1,000) as described previously^53^. The three state covariates with a comparatively strict threshold (segment mean 0.3) was used to genotype the CNVs as one of three type (loss, neutral and gain events) across all the samples. It was noted that the multivariate method tends to detect the common deletions with relative small sizes across multiple samples.

### Population differences across population

We first checked the normalized LRR distribution histograms for all 184 deletion CNVs. The grand majority (99%) of deletions had two distinct peaks, representing neutral (around 0) and homozygous deletion (around -1) states, respectively. Only a couple of deletions had a few samples located in the midpoint between -1 and 0, suggesting a lack of heterozygous events. Additionally, it was difficult to define a universal threshold between homo or heterozygous deletions for all deletions, therefore, we decided to categorize all deletion events using one state: homozygous deletion. To use population genetic programs originally developed for SNPs, we manually recoded each 184 deletion CNVs by converting a loss event into “12” or a neutral event into “22”, where “12” represented a homozygous deletion.

The R Function heatmap.2 (http://www.inside-r.org/packages/cran/gplots/docs/heatmap.2) was used to graph the segment mean LRR values and generate hierarchical cluster dendrograms using 257 CNVs for all animals. We then performed multidimensional scaling (MDS) and admixture analysis to determine how 205 unrelated individuals were clustered according to these CNV genotypes. Using a total of 184 deletion CNVs, MDS analysis of pairwise genetic distance (4 dimensions) was used to detect the relationship between populations with PLINK 1.07 (-mds -plot 4). For a separate verification, we also performed the cluster analysis based on mean LRR values using prcomp functions in R v13.1, the results were consistent with MDS analysis.

Population structure was examined using STRUCTURE 2.3^57^,^58^. Each admixture analysis was performed using 5,000 replicates and 2,000 burn-in cycles under admixture and allele frequencies correlated models.

Neighbor-joining clustering analysis were performed using PHYLIP 3.69 (http://www.phylip.com/) based on pairwise genetic distance. Pairwise genetic distance (D) between individuals was calculated using PLINK 1.07, where D = 1-[IBS2 + 0.5IBS1)/N], and IBS2 and IBS1 are the number of loci that share either 2 or 1 alleles identical by state (IBS), respectively and the N is the number of loci^59^,^60^. The clustering dendrograms were plotted in Figtree 1.3.1 (http://tree.bio.ed.ac.uk/software/figtree/).

### Gene annotation and PANTHER analysis

We retrieved RefSeq, Ensembl, and xenoRefSeq genes overlapping CNV regions by at least 1 bp, including the 5′ and 3′ untranslated regions, from available UCSC genome browser tracks and annotated CNV regions using custom software (https://github.com/njdbickhart/AnnotateUsingGenomicInfo). We performed enrichment analysis using PANTHER classification system^45^. Only clusters with enrichment scores more than 1 (p-value < 0.05 after the Bonferroni correction for multiple testing) were considered.

### Signatures of Selection

To detect the lineage differentiated CNV events, we calculated *V*_*ST*_ for each CNV as previously described^15^ by using the following equation: (*V*_*T*_ −*V*_*S*_)/*V*_*T*_, where *V*_*T*_ is the total variance in mean LRRs across all individuals and *V*_*S*_ is the average variance in cattle within each breed.

## Additional Information

**How to cite this article**: Xu, L. *et al.* Population-genetic properties of differentiated copy number variations in cattle. *Sci. Rep.* **6**, 23161; doi: 10.1038/srep23161 (2016).

## Supplementary Material

Supplementary Information

Supplementary Table S1

Supplementary Table S2

Supplementary Table S3
