# Supplementary material for: Population-genetic properties of differentiated copy number variations in cattle
Source: Sci Rep. 2016 Mar 23;6:23161. doi: 10.1038/srep23161 (PMC4804293; doi:10.1038/srep23161)

Population-genetic properties of differentiated copy number variations in cattle

Lingyang Xu[1,2,5], Yali Hou[3,5], Derek M. Bickhart[1],Yang Zhou[1,4], El Hamidi abdel Hay[1], Jiuzhou Song[2], Tad S. Sonstegard[1,*], Curtis P. Van Tassell[1] and George E. Liu[1,†]

Table S1. Subspecies, breeds, numbers of animals used in this study.

Breed or species	Acronym	Count	Subspecies	Usage	Feature
Taurine					
Angus	ANG	47	*Bos taurus*	Beef	Founded by Angus doddies and Buchan humlies, Aberdeen Angus breed x English longhorns
Brown Swiss	BSW	24	*Bos taurus*	Dairy	Improved by Austrian Pinzgaur breed
Hereford	HFD	37	*Bos taurus*	Beef	Line One
Holstein	HOL	67	*Bos taurus*	Dairy	Holstein is used to describe North American stock while Friesian denotes animals (dual purposes) of a traditional European ancestry
Indicine					
Brahman	BRM	49	*Bos indicus*	Beef	Guzerat, Kankrej, Gir and Nelore are the founders. Krishna Valley strain contributed less.
Nelore	NEL	34	*Bos indicus*	Beef	The Indian Ongole breed contributed most to the creation of the Nelore
African Breeds					
N'Dama	NDA	24	*Bos taurus*	Multipurpose	African taurine, heat resistance
Sheko	SHK	18	Composite	Multipurpose	Ancient Taurine×Indicine hybrid
**Total**		300			

Population-genetic properties of differentiated copy number variations in cattle

Lingyang Xu[1,2,5], Yali Hou[3,5], Derek M. Bickhart[1], Yang Zhou[1,4], El Hamidi abdel Hay[1], Jiuzhou Song[2], Tad S. Sonstegard[1,*], Curtis P. Van Tassell[1] and George E. Liu[1,†]

Figure S1. Box-and-whisker plots of V_{ST} values in four pairwise comparisons.

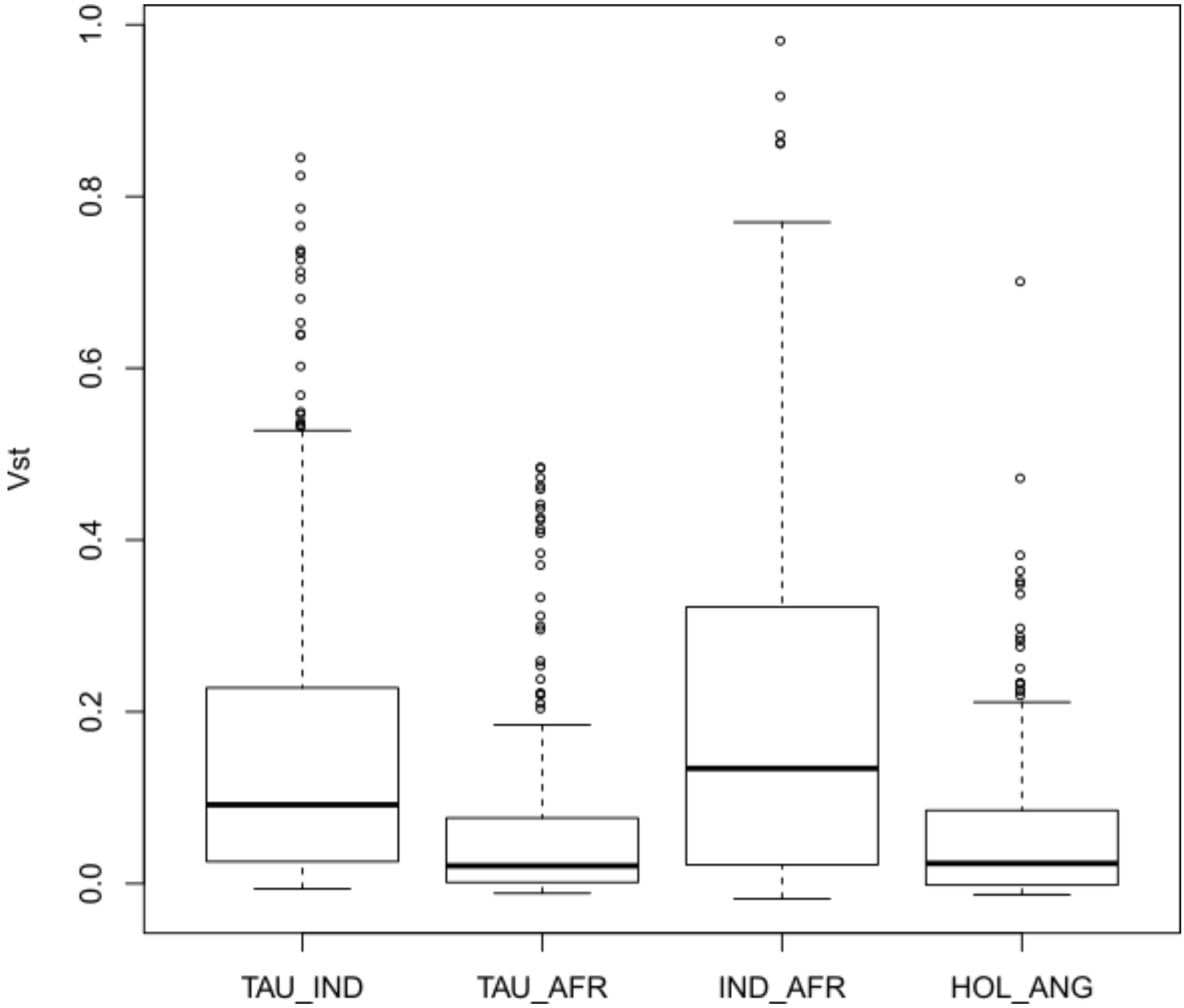

Population-genetic properties of differentiated copy number variations in cattle

Lingyang Xu[1,2,5], Yali Hou[3,5], Derek M. Bickhart[1], Yang Zhou[1,4], El Hamidi abdel Hay[1], Jiuzhou Song[2], Tad S. Sonstegard[1,*], Curtis P. Van Tassell[1] and George E. Liu[1,†]

Figure S2. Violin plots of Log R ratios in eight cattle breeds.

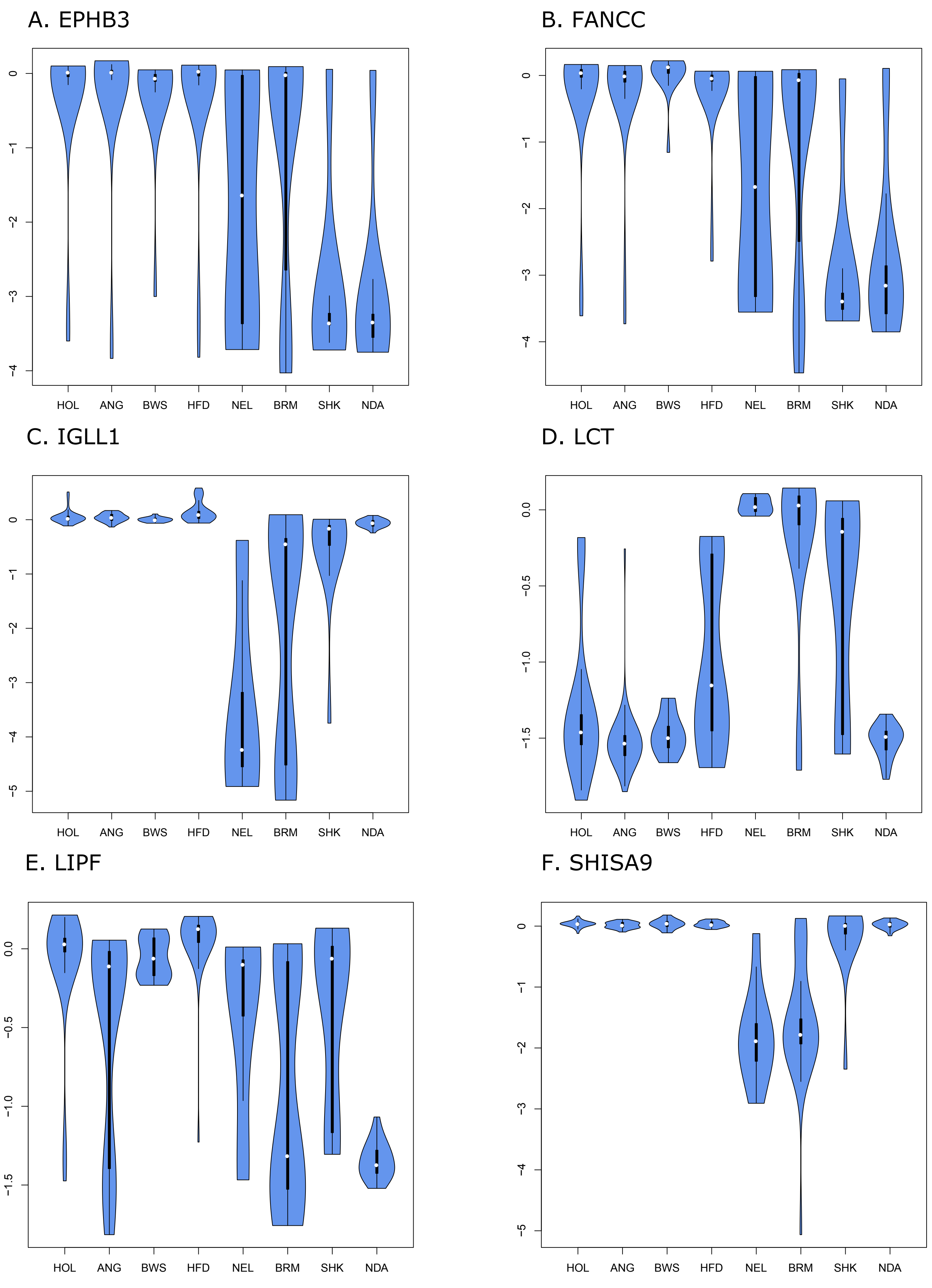

Supplement: Supplementary Information [file srep23161-s1.pdf]
